# Turning genes into medicines—what have we learned from gene therapy drug development in the past decade?

**DOI:** 10.1038/s41467-020-19507-0

**Published:** 2020-11-16

**Authors:** Katherine A. High

**Affiliations:** 1 Perelman School of Medicine of the University of Pennsylvania, Philadelphia, PA 19104 USA; 2 Rockefeller University, New York, NY 10065 USA

**Keywords:** Drug discovery, Biologics, Gene therapy

## Abstract

Gene and cell therapy products approved over the past decade in Europe and North America have provided new therapeutic options for single gene disorders and for hematologic malignancies. Lessons learned, and limitations identified, are reviewed.

The first clinical trial of gene therapy, for a rare inherited form of immunodeficiency, began at the US NIH in 1990; the first approval of a gene therapy drug by a European or North American regulatory agency occurred in 2012, when the European Medicines Agency granted conditional approval for Glybera®, an AAV drug indicated for treatment of a rare lipid disorder, lipoprotein lipase deficiency (https://www.ema.europa.eu/en/medicines/human/EPAR/glybera). Although the launch of the drug was a disappointment, which underscored that commercialization (and key issues of pricing, reimbursement and access) of one-time treatments for rare genetic disorders would require considerable ingenuity and planning, nonetheless successful registration, albeit with extensive post-marking requirements, demonstrated that gene therapy products could in fact win regulatory approval. This helped to unleash a new wave of capital investment in gene therapy; a number of companies exclusively devoted to gene therapeutics were formed, and activity in the space began to expand, gradually at first, and more dramatically over the last few years (Fig. 1). It is thus an opportune moment to look back at what has been accomplished, consider how gene therapy fits into the current therapeutic armamentarium, and analyze from both a scientific and a process standpoint, what we have learned from the successes and the failures of the last decade.

**Fig. 1:**
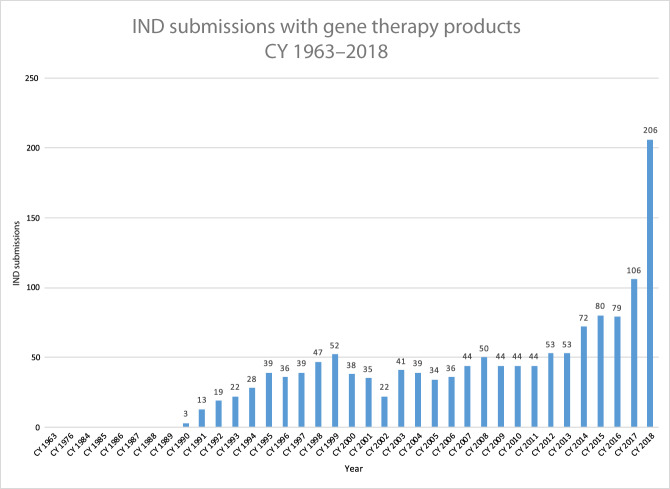
Investigational new drug applications (INDs) in gene therapy by year to US FDA. CY calendar year. Reprinted with permission from Dr. Raj Puri.

There are currently four approved gene therapy products for genetic disease and two products for hematologic malignancies, with many more in development in both categories. From a therapeutic standpoint, the products for genetic disease fall broadly into two classes: those for diseases that previously lacked any pharmacologic treatment, and those for diseases that have traditionally been treated using complex medical regimens frequently characterized by significant non-adherence due to the burden of treatment. The products for hematologic malignancies are for relapsed or refractory B-cell acute lymphoblastic leukemia or for relapsed or refractory B cell lymphomas. This Comment will focus on the products for genetic disease, and will incorporate discussion of both approved and investigational products.

The first approved therapy for a genetic disease in the US (2017) was an AAV product for the treatment of a rare form of congenital blindness caused by autosomal recessive mutations in the gene *RPE65*^1^. With over 250 genes involved in visual pathways, this would seem to be a field ripe for more therapeutic development, and indeed trials are now underway for at least 10 other genetic causes of blindness (https://www.fightingblindness.org/clinical-trial-pipeline). The second AAV product approved in the US (2019), Zolgensma®, is for spinal muscular atrophy (SMA), the most common inherited cause of death in infants^2^. Two other products that utilize either retroviral or lentiviral transduction of autologous hematopoietic stem cells (HSCs) have been approved in Europe but not in the US, Strimvelis® (2016) for the same rare inherited immunodeficiency disorder (due to adenosine deaminase deficiency (https://www.ema.europa.eu/en/medicines/human/EPAR/strimvelis) [ADA-SCID]) addressed by the initial NIH trial, and Zynteglo® (2019) for transfusion-dependent β-thalassemia (TDT) (https://www.ema.europa.eu/en/medicines/human/EPAR/zynteglo). A common feature of all the clinical development programs for these initial gene therapies was approval based on trials that involved relatively small numbers of patients. While this is often the case for therapeutic trials in the setting of rare diseases, in all of these gene therapy trials, the clinical results were clear cut, even dramatic, underscoring the therapeutic potential in the setting of single gene disorders of supplying the missing or defective gene to the affected target tissue. Of these new products, only Luxturna® for RPE65- associated retinal dystrophy falls squarely into the category of providing treatment for a disease that previously lacked any pharmacologic therapy. The other three offer alternatives to existing products or therapeutic regimens, and two (Zolgensma® and Zynteglo®) were approved so recently that it is still too early to know how uptake will compare to other therapies. For both ADA-SCID and TDT, bone marrow transplant from an HLA-matched related donor is still preferred to transplantation of gene-modified autologous cells, but only a minority of patients have a suitable donor. Advantages of the gene therapy approach—an autologous transplant means that the “donor” cells are always available, and graft-versus-host disease is unlikely to occur—are counterbalanced by the need, in thalassemia, for myeloablative conditioning and a several week hospitalization requiring skilled management post-transplant. Once engraftment occurs however, the circulating hemoglobin begins to rise, and patients are freed from lifelong dependence on a regimen of monthly red blood cell transfusions coupled with iron chelation, which have long formed the backbone of medical management. Long-term studies will still be required in order to assess the durability of responses for all these treatments, but the ability to undergo a brief period of intensive medical management, followed by attainment of a new health status that dramatically reduces the need for medical intervention and management, would seem to be an important therapeutic advance for patients born with serious genetic diseases.

A few other lessons arise from analyses of these first four approved products for genetic disease. First, the key strategic insight that characterizes successful programs is the choice of a disease target where current gene therapy can deliver a therapeutic outcome. A thorough understanding of the disease, the vector and its delivery, and requisite levels of transgene expression are necessary but rarely sufficient, and can be deemed the “magic” that underlies the initial successes. In terms of key tactical considerations, development of treatments for diseases that have never had a pharmacologic therapy may necessitate additional work for the drug developer if novel clinical endpoints must be designed, tested and validated^3^ (https://www.fda.gov/regulatory-information/search-fda-guidance-documents/human-gene-therapy-retinal-disorders). This may well be a recurring theme if gene therapy is to fulfill its promise of providing treatments for diseases that have hitherto been untreatable. Third, it may be challenging to utilize the gold standard randomized controlled trial design for lethal diseases that lack any treatment, as was the case for SMA when the first SMA gene therapy trials began. Fortunately in some settings, including SMA and TDT, the natural history data are robust, and the clinical findings in the trials provided clear and unambiguous evidence (for SMA, achieving motor milestones never seen in affected infants; for TDT, achieving transfusion independence) of therapeutic effect. In other settings where trials are still ongoing, for example hemophilia, each patient can serve as his own control using data gathered in a “run-in” period that precedes the gene therapy intervention. Fourth, in a new class of therapeutics, drug developers must be prepared for the challenges that occur when clinical studies reveal findings that had not been predicted by animal models. This is not unique to gene therapy, but has included for example risks related to the human immune response to viral vectors administered directly into the patient^4^, and the risks of germline transmission of donated gene sequences^5^. Fifth, early intervention in genetic disease generally leads to the best therapeutic outcomes, but raises the difficult issue of including children in early clinical testing. In some instances, e.g. SMA, where disease is lethal in early childhood, inclusion of young children is the only possibility. As more experience is gained with a class of therapeutics, and the safety database expands, some of these issues may be resolved.

Although there are now a handful of products approved based on successful trials, there are also several trials that have failed. What lessons can be learned from these? At least some of these have been for complex acquired disorders, e.g. congestive heart failure^6^ (NCT # NCT00454818) and the first trial for age-related macular degeneration^7^ (NCT# NCT01494805). A challenge for this class of diseases is that they often lack large animal models that closely model human disease in terms of etiology or progression; these have been critical to success in gene therapies for monogenic disease, by providing crucial information on starting and efficacious doses, optimal routes of vector administration, and stages at which disease is no longer reversible. A second complexity is the heterogeneity of the clinical population in these disorders, which may complicate or obscure interpretation of trial findings, and a third is the requirement for much larger numbers of participants in trials, if the effect size is modest. Eventual success of gene-based strategies for complex acquired disorders seems certain, especially if investigators analyze and course-correct based on earlier failures, but it is perhaps not surprising that the initial products in gene therapy, with the exception of CAR-T cells for B cell malignancies, have all been for single gene disorders.

No discussion of the past decade in gene therapy is complete without noting the advances that have taken place in both regulatory science and regulatory policy related to gene and cell therapy (GCT). In the US, GCT clinical investigation was for years subject to a dual review system by both the NIH Recombinant DNA Advisory Committee and the FDA. In 2018 the NIH and FDA sought to reduce this duplicative oversight by placing review of investigational drug activity solely in the hands of the FDA, as occurs for other classes of therapeutics^8^. A second key advance has been the development, publication in draft form (2018), and finalization (2020) of a series of guidance documents specific to gene therapy by the US FDA (https://www.fda.gov/vaccines-blood-biologics/biologics-guidances/cellular-gene-therapy-guidances). The work of identifying problems, considering and evaluating the universe of potential solutions, and fixing on generally accepted approaches represents a collaborative effort by sponsors, investigators and regulators. The time and effort involved in developing a regulatory framework and accompanying methodology for novel classes of therapeutics is easy to underestimate, but is a critical hurdle in bringing new therapeutics through to regulatory approval.

A challenge for regulators worldwide has been the dramatic increase in the number of GCT trials under active management (Fig. 1). Given the rapid growth of the field, many of these applications come from entities with limited experience in GCT. It is likely that increased resources for regulatory agencies will be required if the field is to move forward apace.

As activity in the GCT space has expanded, other key limitations have come to light, including the limited worldwide GCT manufacturing capacity; uncertainty over the features that make one product different from another as related to orphan product designation; and the limited number of personnel with training and experience in GCT drug development. These constraints too will need to be addressed.

Nonetheless it is clear that GCT, for all its checkered past, is now giving rise to treatments for diseases that have not previously been treatable, and to life-altering treatments for diseases that had previously demanded Herculean efforts from the patient and the family. Carlota Perez, an economist who studies the effects of innovations on economic development, noted that “…the full fruits of technological revolutions” [are] “only reaped with a time lag. Two or three decades of turbulent adaptation and assimilation elapse from the moment when the set of new technologies, products, industries, and infrastructures make their first impact to the beginning of a ‘golden age’ or ‘era of good feeling’ based on them”^9^. Although it may be difficult in the midst of turbulent adaptation to predict how gene and cell therapies will evolve, the current limited set of approved products represents solution of a wide-ranging series of scientific, technical and medical challenges. Promising clinical trial results in diseases like sickle cell anemia and hemophilia, and the extraordinary amount of activity in the space, suggest that the small number of approved products is likely to grow, in a manner similar to that seen with monoclonal antibodies through the 1990s. Continuing innovations in gene delivery and gene editing will likely expand therapeutic possibilities to encompass complex acquired as well as single gene disorders, as the quest to turn genes into medicines relentlessly gathers momentum.

## References

[1] Russell S (2017). Efficacy and safety of voretigene neparvovec (AAV2-hRPE65v2) in subjects with *RPE65*-mediated inherited retinal dystrophy: a randomised, controlled, open label phase 3 trial. Lancet.

[2] Mendell, J. R. et al. Single dose gene replacement therapy for spinal muscular atrophy. *N. Engl. J. Med.* **377**, 1713–1722 (2017). 10.1056/NEJMoa1706198 29091557

[3] Chung DC (2018). A novel mobility test to assess functional vision in patients with inherited retinal dystrophies. Clin. Exp. Ophthalmol..

[4] Mingozzi F, High KA (2017). Overcoming the host immune response to AAV gene delivery vectors: the race between clearance, tolerance, neutralization, and escape. Annual Rev. Virol..

[5] Boyce N (2001). Trial halted after gene shows up in semen. Nature.

[6] Greenberg B (2016). Calcium upregulation by percutaneous administration of gene therapy in patients with cardiac disease (CUPID 2): a randomised, multinational, double-blind, placebo-controlled, phase 2b trial. Lancet..

[7] Constable IJ (2016). Phase 2a randomized clinical trial: safety and Post Hoc analysis of subretinal rAAV.sFLT-1 for wet age-related macular degeneration. EBioMed..

[8] Collins F, Gottlieb S (2018). The next phase of human gene therapy oversight. NEJM.

[9] Perez C (2002). Technological Revolutions and Financial Capital.

